# Accessory Vertebral Artery: An Embryological Review With Translation from Adachi

**DOI:** 10.7759/cureus.13448

**Published:** 2021-02-19

**Authors:** Stephen J Bordes, Joe Iwanaga, Sina Zarrintan, Koichi Watanabe, R. Shane Tubbs

**Affiliations:** 1 Department of Anatomical Sciences, St. George's University School of Medicine, St. George's, GRD; 2 Department of Neurosurgery, Tulane University School of Medicine, New Orleans, USA; 3 Cardiovascular Research Center, Tabriz University of Medical Sciences, Tabriz, IRN; 4 Department of Anatomy, Kurume University School of Medicine, Kurume, JPN

**Keywords:** accessory vertebral artery, buntaro adachi, embryology, vascular surgery

## Abstract

The vertebral arteries arise from the posterior superior aspect of the bilateral subclavian arteries and course superiorly through the transverse foramina of C1-C6 vertebrae before joining one another along the anterior surface of the pons. Developmental variations during the fourth to sixth weeks of embryonic development may result in the formation of accessory vertebral arteries, i.e., ipsilateral vertebral arteries of dual origin. This anatomical variation is distinct from and often confused with vertebral artery duplications and fenestrations. This article reviews the anatomy and embryology of the accessory vertebral artery with excerpts from Buntaro Adachi’s classic text on vascular anatomical variations. Knowledge of accessory vertebral vessels is important during vascular and spinal procedures of the head and neck. Furthermore, these variations have been associated with cerebrovascular pathologies, such as stroke, dissection, and other hemodynamic anomalies.

## Introduction and background

The vertebral arteries commonly originate as the first and largest branches of the left and right subclavian arteries [1-4]. The arteries extend superiorly, traveling anterior to the C7 transverse process prior to coursing predominately within the transverse foramina from the C1-C6 vertebrae [1,2,5]. The arteries give off various branches and then fuse together, forming the basilar artery anterior to the pons, after passing through the posterior atlantooccipital membrane, dura mater, and foramen magnum [1,2]. These vessels are major arteries of the head and neck, forming the vertebrobasilar system and thus supplying the spinal cord, brain stem, cerebellum, and posterior cerebrum. Anatomical knowledge of these arteries and their variations is crucial during surgical procedures involving the head, neck, and superior mediastinum as injury could lead to hemorrhagic and neurovascular complications. 

**Figure 1:**
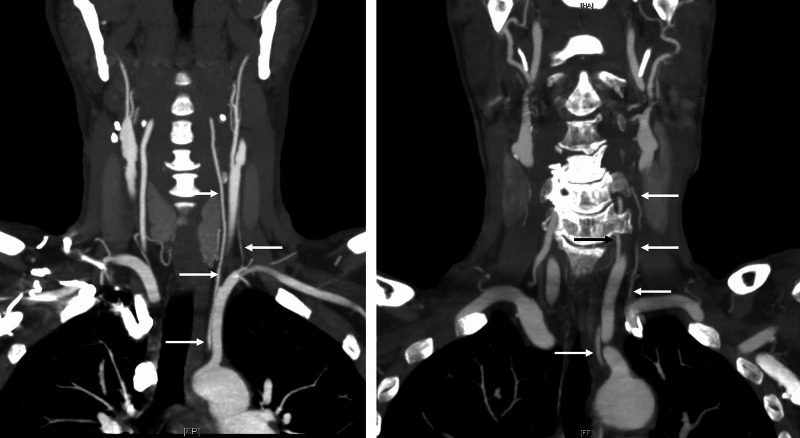
Coronal CTA of two patients with accessory vertebral arteries (right arrows) Both patients also have an aortic arch origin of the left vertebral artery (left arrows). CTA: CT Angiogram

**Figure 2:**
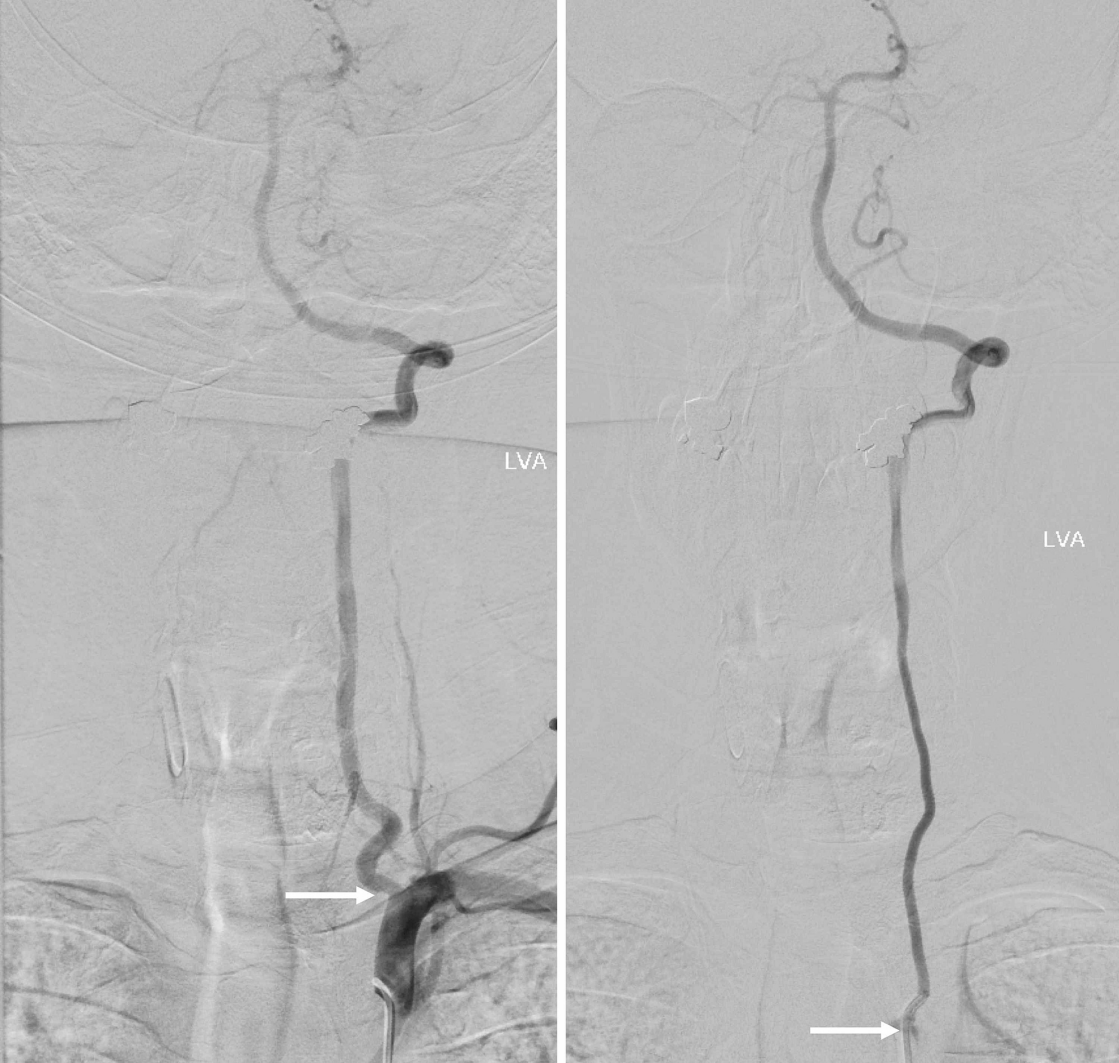
Arteriogram of patient with accessory vertebral artery (left arrow) and left aortic origin of the left vertebral artery (right arrow) Note that both arteries feed into the intracranial blood supply indicating an anastomosis between the two vessels.

**Figure 3:**
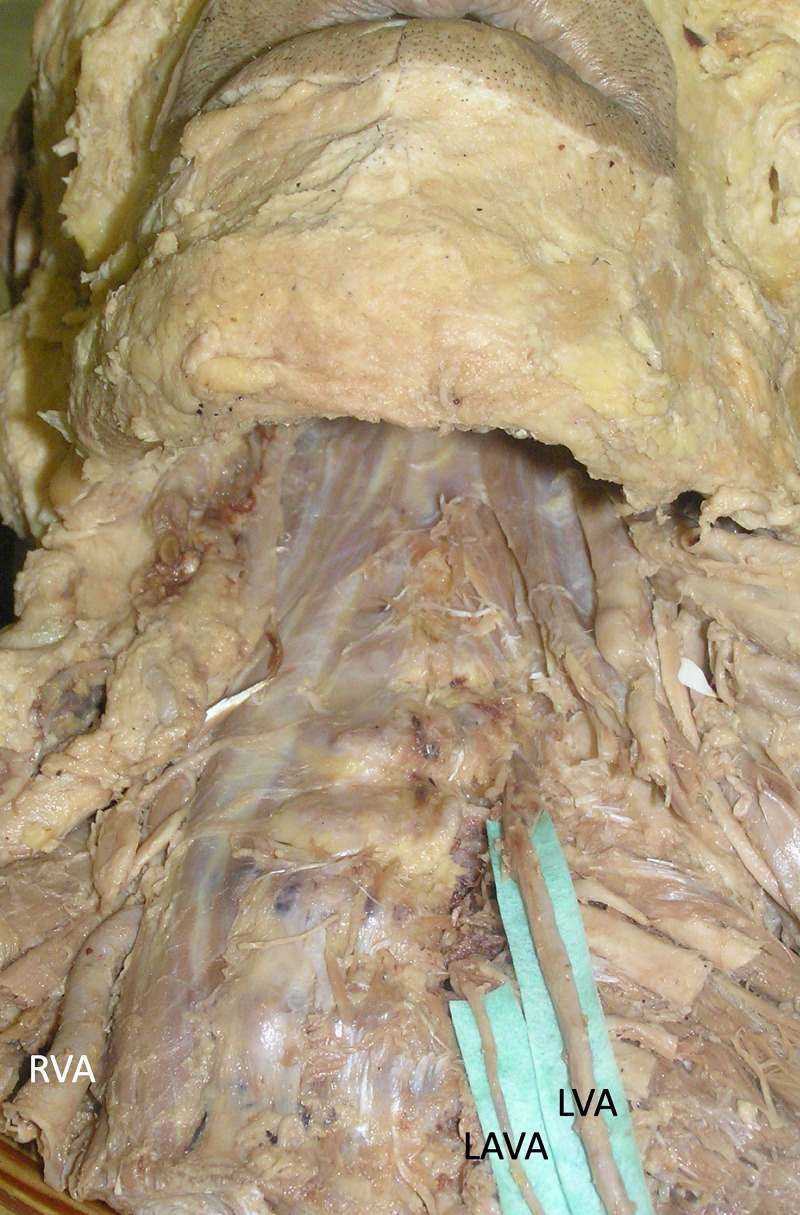
Cadaveric specimen noting a normal right vertebral artery (RVA) and a left accessory vertebral artery (LAVA) with aortic arch origin of the left vertebral artery (LVA)

Buntaro Adachi (1865-1945), a renowned Japanese physician and anatomist who researched human vascular variation, was one of the first to fully describe a variant known as the accessory vertebral artery (Figures 1-3) in his work "Das Arteriensystem der Japaner" (1928) [6-8].

**Figure 4:**
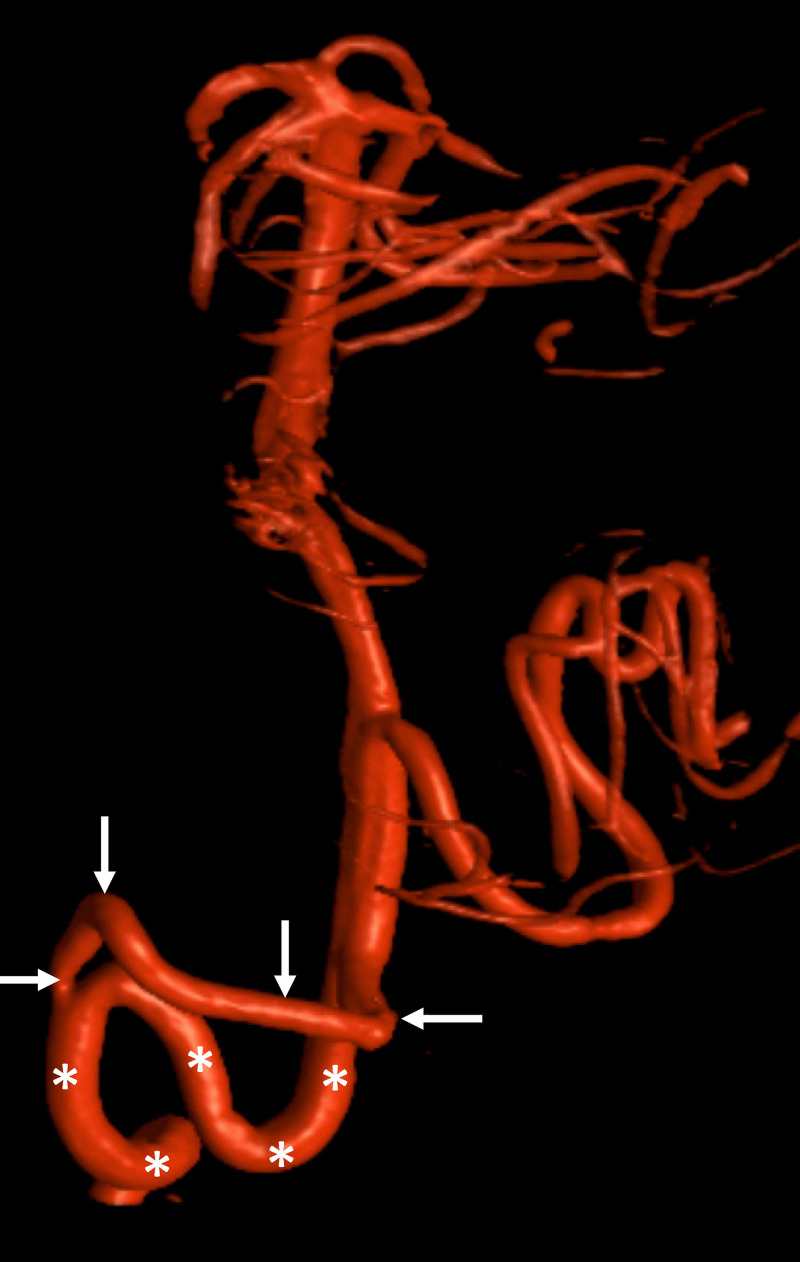
CTA noting a duplicated vertebral artery (arrows) with normal vertebral artery (asterisks) CTA: CT Angiogram

**Figure 5:**
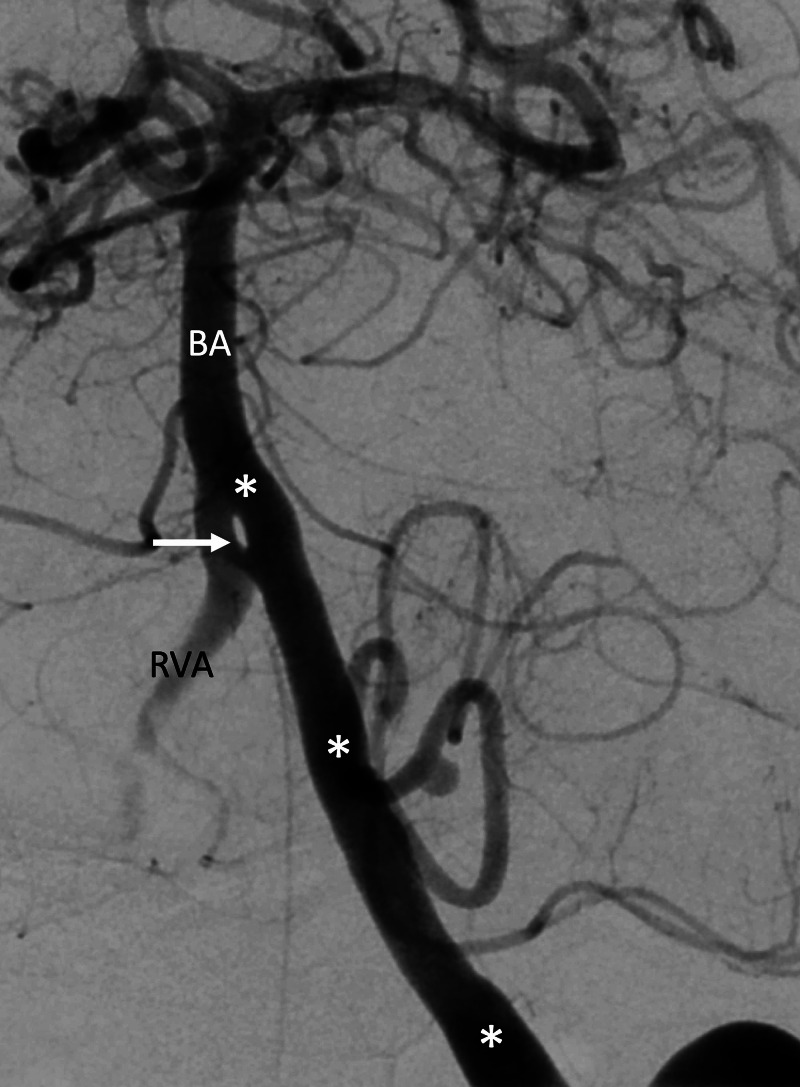
Arteriogram noting a fenestrated left vertebral artery (arrow) at the vertebrobasilar junction The left vertebral artery is noted with asterisks. Also, note the basilar artery (BA) and right vertebral artery (RVA)

The accessory vertebral artery, not to be confused with duplicated (Figure 4) or fenestrated vertebral arteries (Figure 5), are ipsilateral vertebral arteries of dual origin [9].

Adachi mentioned that the accessory vessel typically exists in the presence of a left main vertebral artery that originates from the aortic arch [8]. The accessory artery usually joins the main vertebral artery superior to the level of the C6 vertebra [10]. It may be rudimentary or hypoplastic [4,8,10-14]. However, duplicated vertebral arteries are less frequently observed [10]. This variation results in a vertebral artery of single-origin that later divides into two parts: a typical transverse foraminal segment and an intradural segment [2,9,13,15-20]. As mentioned above, fenestrated vertebral arteries are partially duplicated arteries and as a result, are often confused with vertebral artery duplications [9,17,21]. This variant vessel has a single origin and later divides into two channels, as opposed to two discrete vessels, that are separated by a fenestrated septum [9,17,21]. This variant usually maintains a normal course as it ascends through the transverse foramina. In this review, we provide a translation of Adachi’s “Rudiment of the Common Vertebral Artery,” discuss the embryological origins of the accessory vertebral artery, and compare this accessory vessel with vertebral artery duplications and fenestrations in order to clarify these terminologies.

## Review

Buntaro Adachi’s “Rudiment of The Common Vertebral Artery in the Presence of the Variant Vertebral Artery” (translated from German)

In the specimen with a variant vertebral artery, more often a fine artery can be found that is regarded as a rudiment of the common vertebral artery. It originates from the subclavian artery and passes through the transverse foramen of the sixth cervical vertebra. I have recorded this rudimentary artery only occasionally. I have already illustrated some of the preparations.

**Figure 6:**
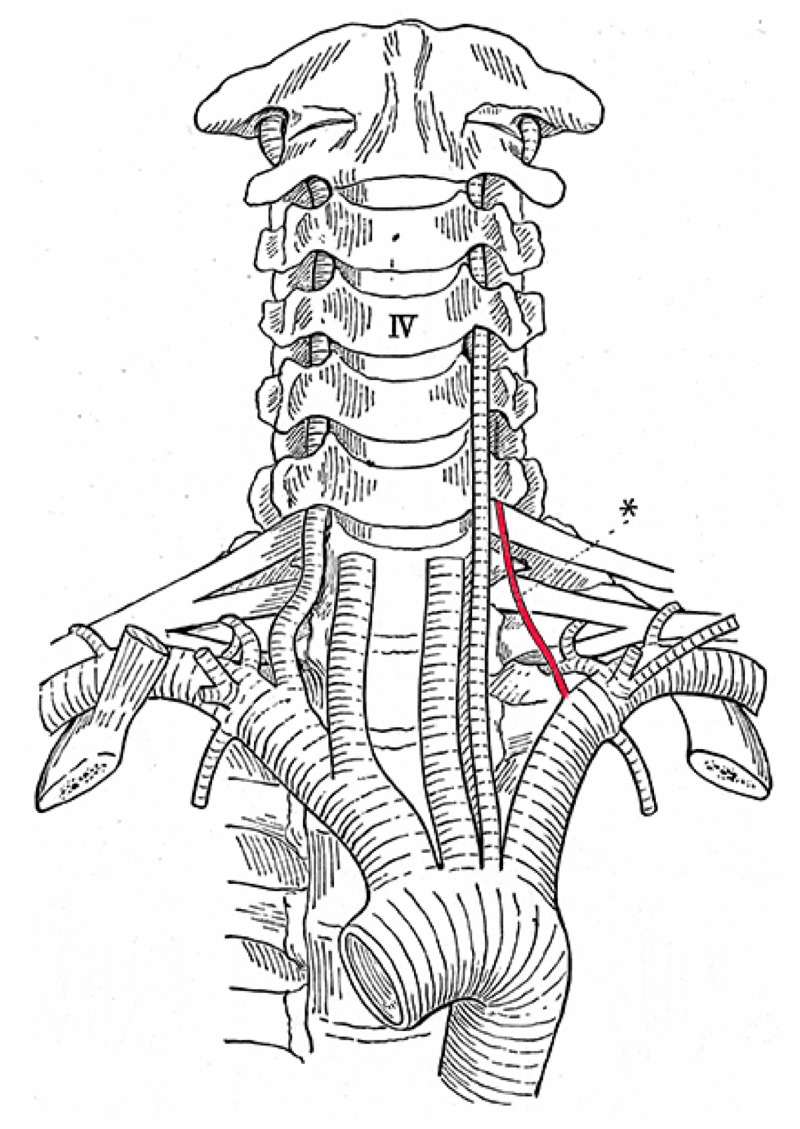
Left accessory vertebral artery (asterisk) and left-sided aortic arch origin of the left vertebral artery (After Adachi)

In Figure 6, nothing more is to be highlighted except that from the left vertebral artery originating from the aortic arch and entering the fifth transverse foramen, there is a rudimentary normal artery.

**Figure 7:**
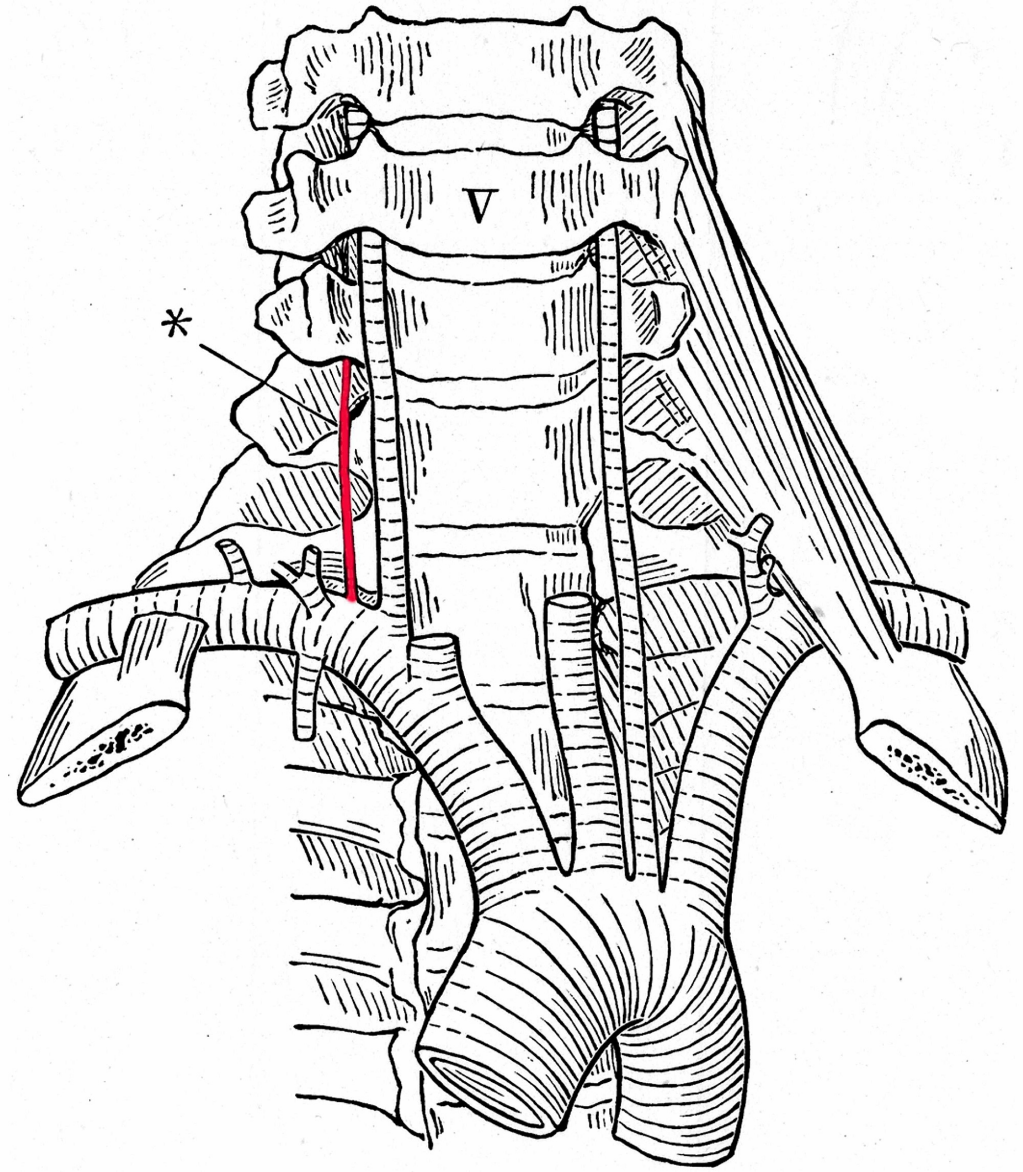
Right accessory vertebral artery (asterisk) with left-sided aortic arch origin of the left vertebral artery (After Adachi)

Figure 7 already proves that the origin of the right vertebral artery, passing the fifth cervical vertebra, is moved proximally and therefore closer to the angle of the brachiocephalic artery than the one of the normal artery.

**Figure 8:**
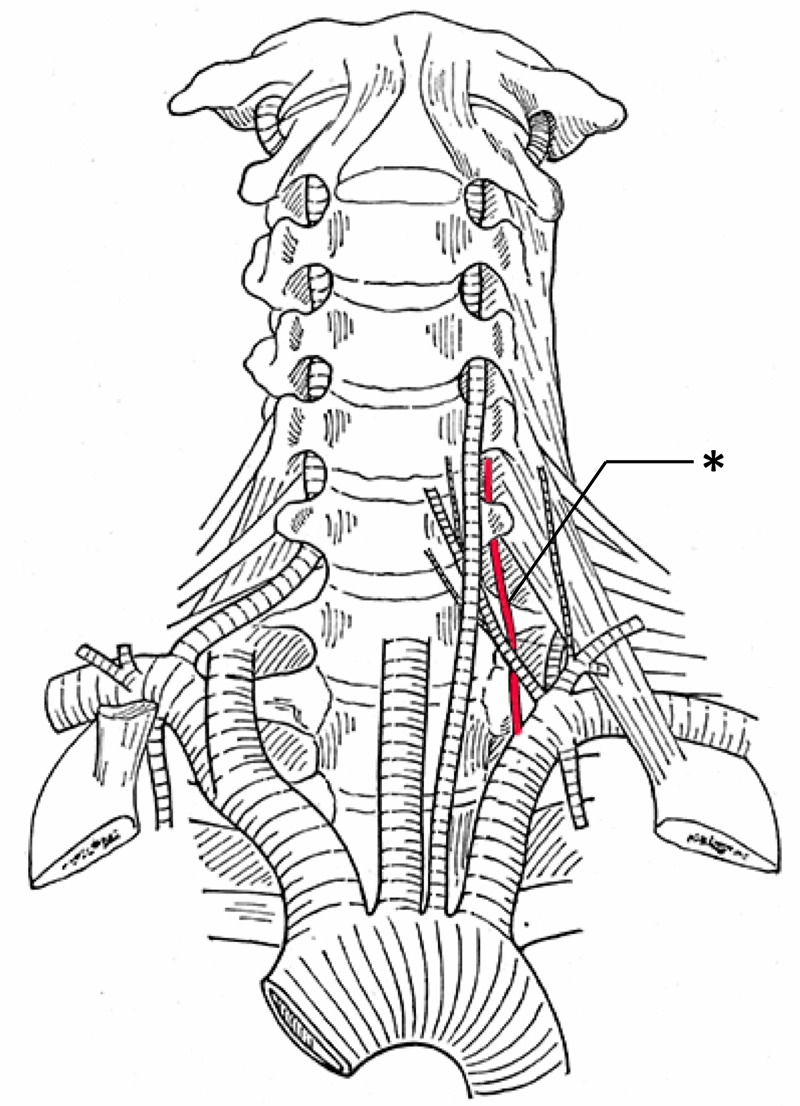
Left accessory vertebral artery (asterisk) and left-sided aortic arch origin of the left vertebral artery (After Adachi)

I have already emphasized that the specimen shown in Figure 8 is very instructive. The strongly formed abnormal left vertebral artery courses in front of the inferior thyroid artery; the normal, however rudimentary one, behind the inferior thyroid artery.

**Figure 9:**
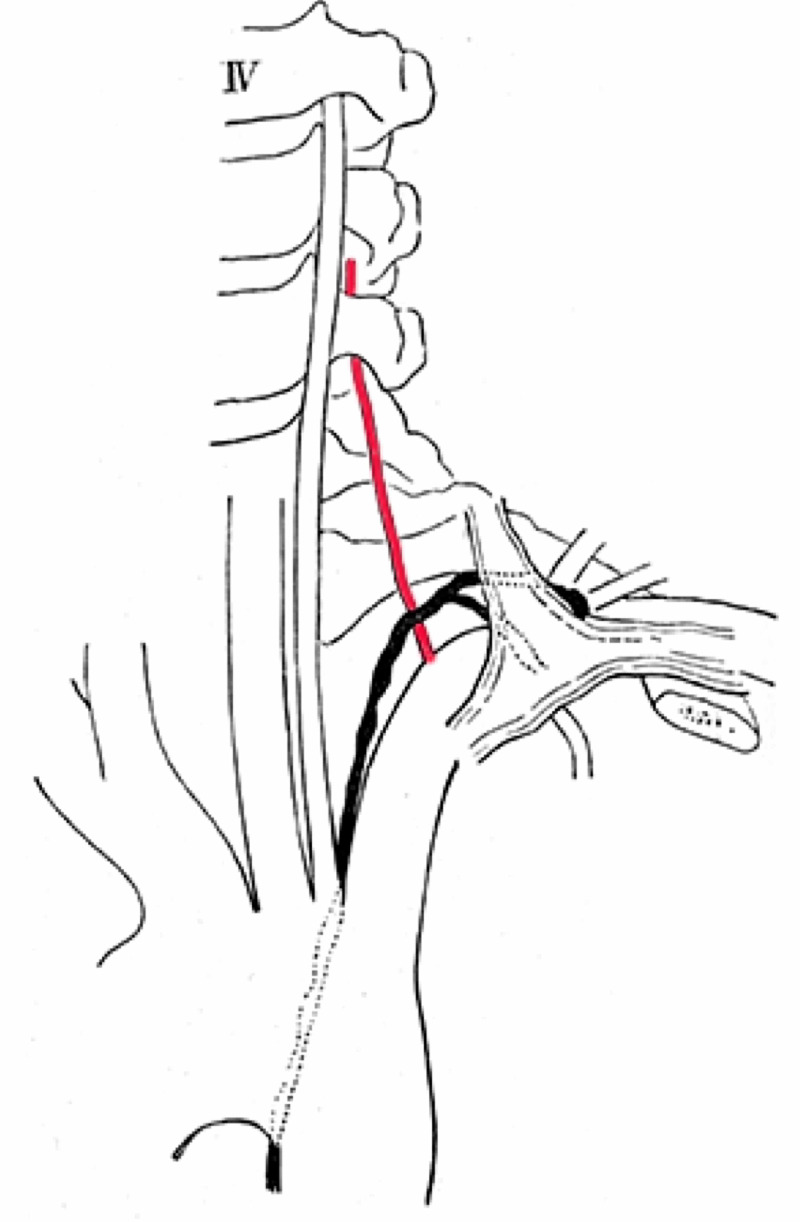
Left accessory vertebral artery (red) and left-sided aortic arch origin of the left vertebral artery. Note the relationship between the accessory vertebral artery and thoracic duct colored in black (After Adachi)

Similarly, in Figure 9, the left vertebral artery arising from the aortic arch takes an abnormal position toward the thoracic duct while the rudiment of the normal artery is located in the normal position, behind the thoracic duct.

So far, I was unable to prove that the rudiment of the normal artery forms an anastomosis with the strong, variant artery; it always ended very soon after the entry into the sixth transverse foramen. In the European literature, a vertebral artery with two (or three) roots has been reported, and the normal and variant arteries are both well developed and soon unite into a common trunk or an ascending cervical artery anastomosis with a vertebral artery. 

Embryology of the aortic arch and vertebral arteries

**Figure 10:**
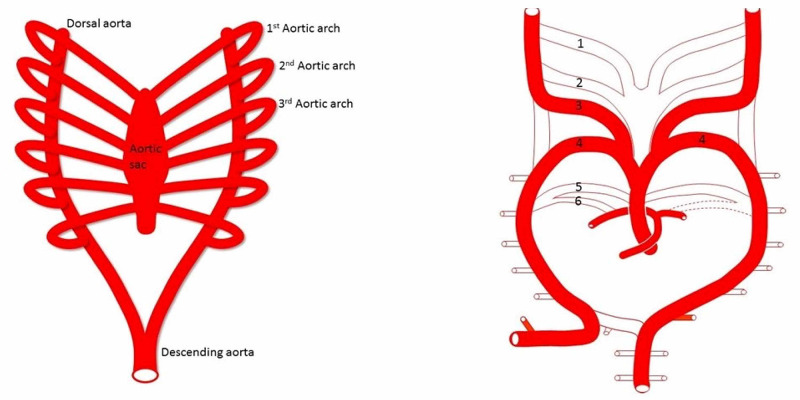
Development of the vertebral artery Left: During the fetal period, along with the development of the pharyngeal arches (not shown), six aortic arches connect the aortic sac and right and left dorsal aortae to supply each pharyngeal arch. Right: The first and second aortic arches regress and almost disappear. The fifth aortic arch becomes a remnant and the sixth aortic arch becomes the pulmonary artery

In order to explain vertebral artery variations, the embryology of the great vessels must first be understood. The aortic arch and its branches are formed from the residual segments of six pairs of embryological aortic arches during the fourth to sixth weeks of development [2,3,22] (Figure 10)

**Figure 11:**
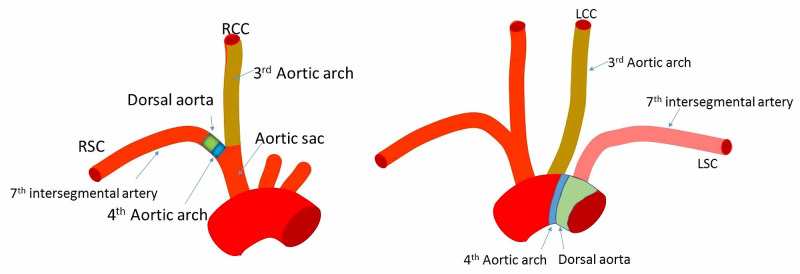
The third aortic arch becomes the right and left common and internal carotid (not shown) arteries The left fourth aortic arch becomes the aortic arch, and the right becomes a part of the subclavian artery. The blue green area (Left) would be the origin of the subclavian artery where the right vertebral artery arises. The blue green area (Right) would be the origin of an aortic arch origin of the left vertebral artery. The left normal vertebral or left accessory vertebral artery would arise from the left subclavian artery here shown in pink as derived from the 7th intersegmental artery. RSC, right subclavian artery; RCC, right common carotid artery; LCC, left common carotid artery; LSC, left subclavian artery

These arches connect the bilateral dorsal aortae and aortic sac [2]. The first and second aortic arches regress rather quickly. The third pair of arches later form the bilateral common and internal carotid arteries [2,3,22]. The fourth pair of arches form the aortic arch on the left and brachiocephalic trunk on the right [2,3,22] (Figure 11)

**Figure 12:**
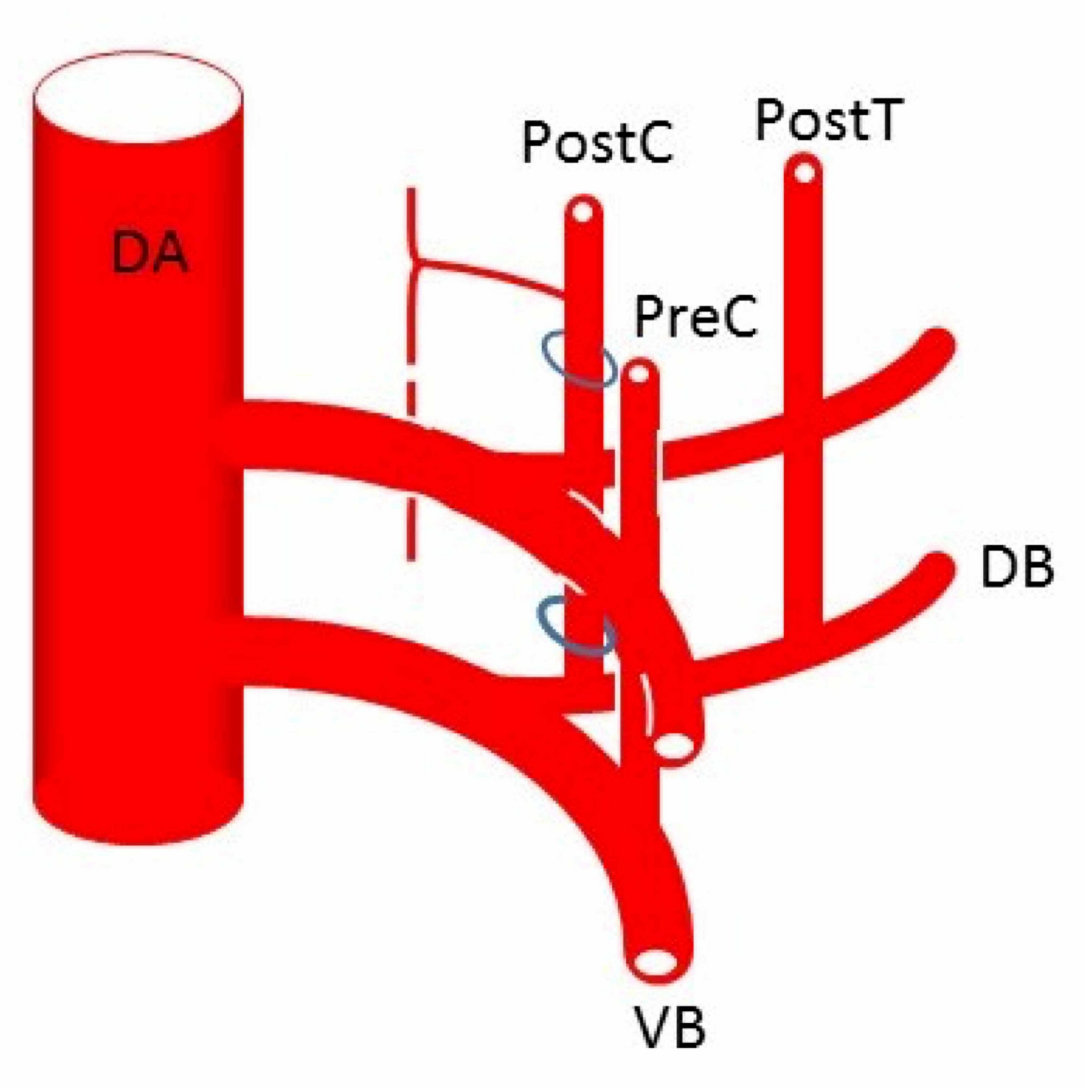
The dorsal aortae (DA) gives rise to dorsal (DB) and ventral (VB) branches Dorsal branches are called intersegmental arteries (30 pairs) and the seventh intersegmental artery becomes the subclavian artery. Intersegmental arteries in the neck usually regress and the upper and lower intersegmental arteries anastomose longitudinally to form new vessels. The vertebral artery is formed by a connection between the postcostal (PostC) anastomosis formed by dorsal branches of the first to sixth intersegmental arteries and seventh intersegmental artery that becomes the subclavian artery. In a similar manner, the thyrocervical trunk and costocervical trunk are formed by precostal (PreC) and posttransverse (PostT) anastomoses, respectively

The fifth arch typically regresses, and the sixth arch forms the right pulmonary artery and ductus arteriosus [14]. The dorsal aorta sends off ventral, lateral, and dorsal branches (Figure 12).

The dorsal branches form dorsal intersegmental arteries which travel with each somite [2,3,22]. The vertebral arteries originate at the ends of the seventh intersegmental arteries, which normally form the subclavian arteries [22]. The remainder of each vertebral artery is created from transverse anastomoses of the first through sixth intersegmental arteries bilaterally [2]. 

Aortic arch origin of a vertebral artery

It is important to note that vertebral artery variation occurs more frequently on the left than the right [22]. Right-sided variants are extremely rare and such may be attributable to the absence of a right aortic arch [3]. A left vertebral artery arising directly from the aortic arch is the most common vertebral artery variation (prevalence ranging from less than 1% to 5.8%) [3,10]. It is believed that persistence of the distal segment of the fifth or sixth intersegmental artery leads to the origination of one or both vertebral arteries from the aortic arch as opposed to their respective subclavian artery [22]. Other theories posit that either the aorta is comprised of a piece of the seventh intersegmental artery or excessive embryological absorption occurs between the aortic arch and subclavian artery [3]. 

Embryology of accessory vertebral arteries

Accessory vertebral arteries arise when multiple segments of the intersegmental arteries persist [2,3]. In other words, one or more intersegmental arteries incompletely regress and maintain a connection between the dorsal aorta and true vertebral artery [3]. As a result, a vertebral artery may have two or more origins that eventually fuse together, most commonly superior to the C6 vertebral level. Adachi found that accessory vertebral arteries always accompany a left vertebral artery of aortic origin [8]. Recent studies have shown that accessory vertebral arteries exhibit great variability and may originate from any of the great vessels or their branches [1,2,10,16,22]. 

Embryology of duplicated vertebral arteries

As mentioned above, vertebral artery duplication refers to the division of a vertebral artery into a normally coursing segment and an intradural segment (Figure 4) [9]. From an embryological standpoint, the intradural segment is the product of an intersegmental artery that would have otherwise formed a radicular artery [9,23,24]. Duplications are regularly found at the C1-C2 vertebral levels and have a prevalence of 0.7% [9,11,13]. Within the vertebrobasilar system, duplications of the basilar artery are most frequently observed [9]. 

Embryology of fenestrated vertebral arteries

A fenestrated vertebral artery (Figure 5) is a product of partial division of the vertebral artery, most often observed superior to the C3 vertebral level [9]. The vertebral artery consists of two channels within the same vessel, most likely due to incomplete regression of intersegmental arteries.

Clinical and surgical significance

**Figure 13:**
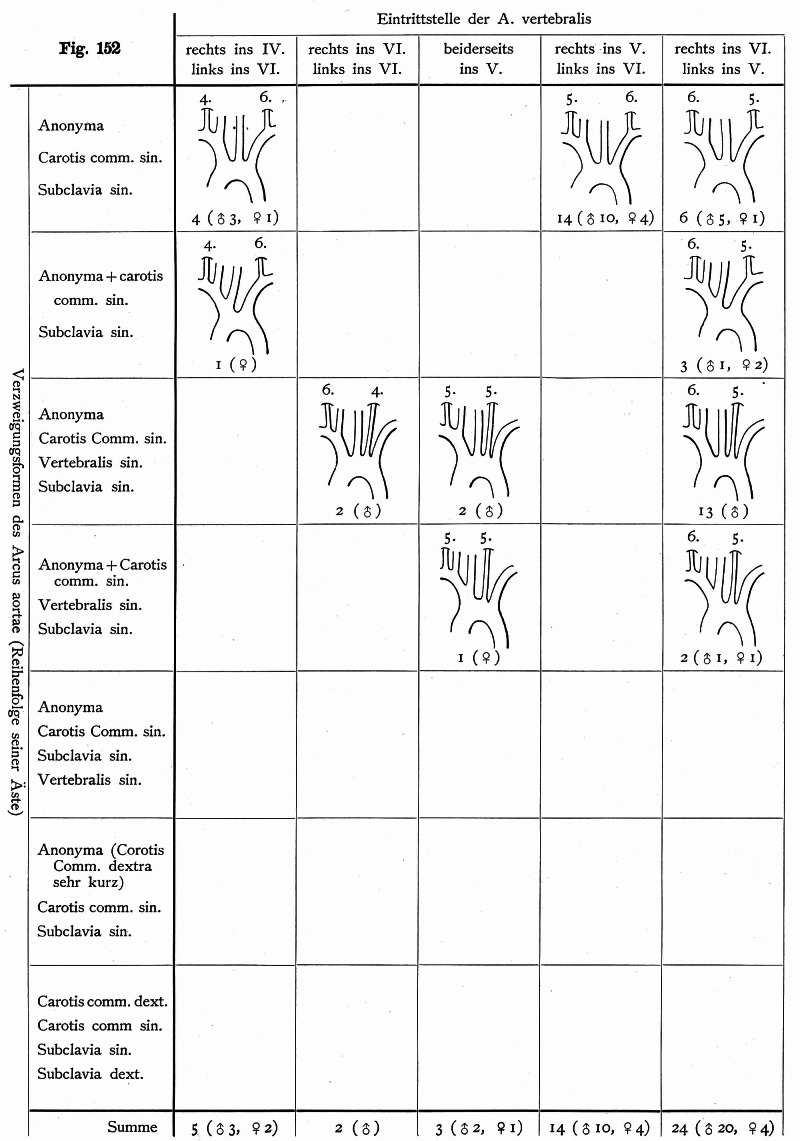
Table from Adachi’s work illustrating, in schematic form, the various accessory vertebral arteries that he documented (After Adachi)

Individuals with vertebral artery variations are generally asymptomatic, but some studies have shown associations between accessory vertebral arteries and individuals with Down syndrome [1,3,4,10]. Symptoms such as dizziness tend to be largely nonspecific and typically stem from other etiologies [3]. Knowledge of vertebral artery course and variation is crucial during surgical procedures of the thorax and neck, especially those requiring anterior cervical spine approaches, cervical spine epidural injection, or vertebral artery stenting [1,3,4,10]. Some studies have indicated that vertebral artery variation leads to alternations in hemodynamics and should be evaluated in cases of cerebrovascular accidents, thromboembolic events, and vertebral artery dissections [3,22]. As such, there is a strong correlation between vertebral artery variation, stroke, and other thromboembolic events. Vertebral artery duplications tend to be clinically silent, but fenestrations have been associated with intracranial pathology such as aneurysms and stroke [9]. Vertebral arteries originating from the aortic arch have a longer course and are thus more prone to dissection as a result of increased shearing force [3,22]. As shown by Adachi, accessory vertebral arteries are usually posterolateral to the main vertebral artery and have a course posterior to the termination of the thoracic duct (Figure 9). These arterial variations are readily detected using CT and MR angiography (Figures 1-2) [22,25]. Imaging should be obtained prior to cervical spine procedures, vascular procedures of the head and neck, and cardiothoracic surgery [3,22]. As highlighted by Adachi, surgeons should be alerted to the possibility of an accessory vertebral artery in the presence of a vertebral artery of aortic arch origin (Figure 13).

## Conclusions

The vertebral arteries are subject to a wide range of vascular variations as they arise from the development and regression of primitive vessels in early embryonic stages. Knowledge of these variants is of the utmost importance during surgical and interventional procedures to decrease the risk of vascular complications and for minimizing misdiagnoses. Furthermore, we emphasize the differences among accessory, duplicated, and fenestrated vertebral arteries in addition to the correlation between vertebral artery variations and cerebrovascular complications such as stroke.
